# Protective effect of rutin on the antioxidant genes expression in hypercholestrolemic male Westar rat

**DOI:** 10.1186/1472-6882-13-136

**Published:** 2013-06-17

**Authors:** Salem S Al-Rejaie, Abdulaziz M Aleisa, Mohamed M Sayed-Ahmed, Othman A AL-Shabanah, Hatem M Abuohashish, Mohammed M Ahmed, Khaled A Al-Hosaini, Mohamed M Hafez

**Affiliations:** 1 Department of pharmacology and toxicology; College of pharmacy, King Saud University, Riyadh, Kingdom of Saudi Arabia

**Keywords:** Hypercholesterolemic liver, Rutin, Oxidative stress genes, Real time PCR

## Abstract

**Background:**

High-cholesterol diet (HCD) increases the oxidative stress in different tissues leading to many diseases. Rutin (RT) is a natural flavonoid (vitamin p), which possesses an antioxidant activity with protective potential. The present study aimed to examine the potential effects of rutin on hypercholesterolemia-induced hepatotoxicity in rat.

**Methods:**

Male Wistar rats were divided into four groups: GI) control (Rat chow), GII) Rutin (0.2% in rat chow), GIII) HCD (1% cholesterol and 0.5% cholic acid in rat chow) and GIV) rutin (0.2%) + HCD.

**Results:**

Rutin in combination with HCD induced a significant protective effect against the hepatotoxicity by reducing the plasma level of alanine transaminase (ALT), aspartate aminotransferase (AST), triglyceride (TG), total cholesterol (TC), and low-density lipoprotein (LDL). The HCD (GII) showed a decrease in glutathione peroxidase (GPx), glutathione reductase (GR) and increase in glutathione S transferase α (GSTα), sulfiredoxin-1(Srx1), glutamate-cysteine ligase (GCL) and paraoxonase-1(PON-1) genes expression levels.

**Conclusion:**

Treatment with rutin reversed all the altered genes induced by HCD nearly to the control levels. The present study concluded that the HCD feedings altered the expression levels of some genes involved in the oxidative stress pathway resulting in DNA damage and hepatotoxicity. Rutin have a hepatoprotective effect through the mechanism of enhancing the antioxidant effect via amelioration of oxidative stress genes.

## Background

Hypercholesterolemia is considered as one of the most familiar metabolic disorders and it is closely associated with obesity, diabetes mellitus, and several other metabolic syndromes [1,2]. It can eventually lead to non-alcoholic fatty liver disease (NAFLD) by depositing the lipids and triglycerides in liver which is usually progress to nonalcoholic steato-hepatitis (NASH), cirrhosis, liver failure and hepatocellular carcinoma [3,4]. NAFLD is characterized by destruction in liver n-6 and n-3 long-chain polyunsaturated fatty acids [5,6]. A major factor associated with these liver fatty acids depletion in obesity is the development of prolonged oxidative stress, which may be compounded by defective desaturation activity and dietary imbalance, promoting hepatic steatosis [6,7]. Earlier studies demonstrated that, even short exposure to HCD is capable of inducing hypercholesterolemia and is significantly associated with oxidative stress [8].

Obesity is increasing worldwide especially in places with high dietary fat intake and is associated with lot of complications including NAFLD [9]. NAFLD is a common disease with an estimated prevalence in unselected population of developed nations around 20–30% [10]. From last three decades, Saudi Arabia has been undergoing massive developments. These developments are causing drastic changes in lifestyles and dietary habitats, like many other developed societies some of these changes tremendously increasing physical disorders such as obesity and NAFLD. The prevalence of overweight in Saudi Arabia is 36.9% and more prevalent in males (42.4%) than females (31.8%) [11] and recently Al-hamoudi et al., reported prevalence of NAFLD is around 17% in Saudi population [12].

Accumulation of lipid in hepatocytes may cause a dysfunction in the synthesis of fatty acids. Transcription factors such as sterol-regulatory-element-binding protein-1c (SREBP) and peroxisome proliferator-activated receptor alpha (PPARs) promote hepatic fatty acid synthesis. Long chain polyunsaturated fatty acids and acyl-CoAs, are metabolic regulators of many transcription factors that motivate the liver lipid metabolism. Fatty acids induce changes in the activity of four transcription factor families: PPARs, LXRs, hepatic nuclear factor 4, and SREBP [13,14]. Downregulation of gene expression by fatty acids would be restricted to polyunsaturated fatty acids, but the upregulation would be independent of the saturation [15]. These Differences might involve differential metabolism (oxidative pathways, kinetics etc.) and selective transport of fatty acids to the nucleus. Polyunsaturated fatty acids regulates the genes involved in fatty acid oxidation such as PPARa target genes in which suppress SREBP-1c activity, leading to a reduction in liver triacylglycerol content [16]. The liver is a major source of newly synthesized cholesterol. Cholesterol can be derived from newly-absorbed cholesterol, peripheral tissues and cholesterol synthesized within liver. Cholesterol taken up by the liver is in the form of cholesterol esters [17] which can be either stored as esters or hydrolyzed to free cholesterol [18].

Oxidative stress is highly correlated with a wide variety of inflammatory, cancer, brain disorders, and metabolic disease states, including obesity [19,20]. It is highly correlated with cumulative damage done by reactive oxygen species (ROS) and reactive nitrogen species (RNS) inadequately neutralized by antioxidants mechanisms [21,22]. It has been shown that free radicals may adversely affect cell survival through the oxidative damage of lipid, protein, and irreversible DNA modification [23,24]. Damage, at the cellular level by oxidants, is attenuated by antioxidant enzyme [25]. Furthermore oxidative damage is aggravated by the decrease in antioxidant enzymes activities which acts as a free radical scavengers in conditions associated with oxidative stress [26]. Superoxide dismutase is one of the major enzymatic antioxidant mechanisms against superoxide radical, prevents liver toxicity induced by oxidative stress [27]. Catalase and GSHPx catalyze dismutation of the superoxide anion (O_2_-) into hydrogen peroxide (H_2_O_2_) which then converting H_2_O_2_ to water thus providing protection against ROS [28]. The reduction in activity of these enzymes may be caused by the increase in free radical induced by HCD [29,30]. Paraoxonase (PON1) is another antioxidant enzyme closely associated with high-density lipoproteins, which detoxifies lipid peroxides, and is widely distributed in many tissues, such as liver [31]. Sulfiredoxin-1 enzyme, belongs to the family of oxidoreductases, catalyzes reduction of cysteine sulfinic acid to sulfenic acid in oxidized proteins and protects them from inactivation [32]. Glutamate–cysteine ligase, composed of catalytic subunit (called GCLC) and regulatory subunit (called GCLM), is important for GSH biosynthesis in combating a variety of oxidative stress-related complications, thereby activating the body’s own protective response [33].

Flavonoids are polyphenolic compounds found in plants and have an important role in detoxification of free radicals [34]. Rutin, flavonoid glycosides, possesses different protective effects such as hepatoprotective against carbon tetrachloride (CCl4)-induced liver injuries in rats, ischemia/reperfusion-associated hemodynamic alteration through antioxidant activity [35-39]. It has an inhibitory effect against membrane lipid peroxidation and oxidative stress-mediated diseases [40]. In order to find out the possible mechanisms mediating the antioxidant effect of RT, the present study evaluate its effect on gene expression of hepatic antioxidant enzymes in male Wistar rats fed with HCD as animal models for NAFLD.

## Methods

### Animals used

Twenty four young male Wistar albino rats six weeks old with average body weight 80–100 gms, were obtained from the Animal Care Center, College of Pharmacy, King Saud University, Riyadh, Saudi Arabia. The animals were acclimatized to laboratory condition prior ten days to the experiment. They were fed on Purina rat chow diet (Manufactured by Grain Silos &Flour Mills Organization, Riyadh, Saudi Arabia) and water ad libitum and were maintained under standard conditions of temperature (22±1°C), humidity (50-55%) and a 12 h light/dark cycles. All methods including euthanasia procedure were conducted in accordance with Guide for care and use of laboratory animals, institute for laboratory animal research, National Institute of Health (NIH publication No. 80–23; 1996) and it has approved by Research Ethics Committee of Excremental Animal Care Center, College of Pharmacy, King Saud University, Riyadh Saudi Arabia.

### Dietary protocol and experimental groups

#### Dietary protocol

Experimental diets were prepared in pellet form by adding 0.2% rutin (RT) [41] (power, sigma, USA) or 1% cholesterol + 0.5% cholic acid (HCD) [42] or 0.2% RT + 1% cholesterol + 0.5% cholic acid (RT+HCD) in rat chow powder. Rat chow was used as normal diets and was prepared weekly and shade dried.

#### Experimental group

The Animals were randomly divided into 4 groups of 6 rats in each as follows:

Group (I): Animals received rat chow only and considered as control.

Group (II): Animals received rat chow + 0.2% rutin (RT)

Group (III): Animals received rat chow + {1% cholesterol +0.5% cholic acid }(HCD)

Group (IV): Animals received rat chow + 0.2% rutin (RT) +{1% cholesterol +0.5% cholic acid } (HCD).

The experimental diets were supplemented for 6 consecutive weeks. During whole experimental period, all groups of animals were kept on free access to food and water. At end of the experiment, animals were sacrificed by decapitation and the trunk blood was collected in heparinized tubes. Liver tissues were rapidly excised, weighed and kept in −80°C until used. Plasma samples were collected after centrifugation at 1252 g for 15 min and kept in −20°C until used.

#### A-Bioassay measurments

I- Blood chemistry

Plasma levels of aspartate aminotransferase (ALT), alanine aminotransferase (ALT), total cholesterol (TC), triglyceride (TG), high density lipoprotein (HDL), and low density lipoprotein (LDL) were estimated by using commercially available diagnostic kits (Human, Wiesbaden, Germany).

II- Estimation of Malondialdehyde (MDA) in liver

The method described by Ohkawa et al., [43] was used to determine MDA concentration in liver. Briefly, 200 mg of liver tissues were homogenized in aqueous 0.15M KCl solution to give 10% homogenate. One ml of homogenate was then mixed with one ml of 10% trichloroacetic acid (TCA) and centrifuged at 704 g for 15 min. one ml of supernatant was suspended into one ml of 0.67% 2-thiobarbutaric acid. Sample tubes were then placed into a boiling water bath for 15 min. Samples were allowed to cool down at room temperature followed by centrifugation at 704 g for 15 min. The optical density of the clear pink supernatants was measured at 532 nm by using spectrophotometer (LKB-Pharmacia, Mark II, Ireland).

III- Estimation of (Glutathione) GSH levels in liver

The concentration of GSH was determined as described by Sedlak and Lindsay [44]. Briefly, 200 gm from liver tissue were dissected out and homogenized in ice-cold 0.02M ethylenediaminetetraacetic acid (EDTA). An aliquots of 0.5ml of tissue homogenate was mixed with 0.2M Tris buffer, pH 8.2 and 0.1 ml of 0.01 M Ellman’s reagent, [5,5’-dithiobis-(2-nitr-benzoic acid)] (DTNB). Each sample tube was centrifuged at 704 g at room temperature for 15 min the absorbance of the clear supernatant was measured using spectrophotometer (LKB-Pharmacia, Mark II, Ireland) at 412 nm.

IV- Assessment of plasma hydrogen peroxide concentration

Plasma H_2_O_2_ concentration levels were measured by BioVision assay kit (BioVision, CA, USA). The principles based on the present of horse radish peroxidase, the OxiRed probe react with H_2_O_2_ to produce product with color that can be measure.

#### B- Assessment of gene expression level (GPx, GR, Srx1, GCL, PON-1 and GST-α genes) by real time PCR in liver tissues

I- Total RNA extraction

Total RNA were extracted from liver using RNA Mini kit (Bioline, Taunton,USA) according to the manufacturer's protocol. The quantity and integrity of total RNA were characterized using a UV spectrophotometer (Nanodrop 8000 Thermo Scientific, USA) and ethidium bromide stained agarose gel. The isolated RNA has an A 260/280 ratio of 1.9–2.0.

II- cDNA synthesis and real time PCR methods

First-strand cDNA was synthesized from 1μg of total RNA by reverse transcription with a SuperScript™ first-strand synthesis system kit (Invitrogen, CA, USA), according to the manufacturer's instructions. Real time PCR using ΔΔCT method was done according to previous study [45]. We used GAPDH gene as housekeeping gene. All primers used in this study were synthesized in Metabion Company (Metabion international AG, Germany) and listed in Table 1.

**Table 1:** Showing primers and probe used

**Gene name**	**Forward primer**	**Reverse primer**	**Probe**
Glutathione peroxidase	5’-GGTGTTCCAGTGCGCAGAT-3’	5’-AGGGCTTCTATATCGGGTTCGA-3’	FAM-CAGCAGGCGCTTTCGCACCA-TAMRA
Glutathione reductase	5’-TGAGCCGCCTGAACAACA-3’	5’-TTGCGTAGCCGTGGATGAC-3’	FAM-CCAAAACAATTTAACCAAGTCCCACATCGA-TAMRA
Glutathione s transferase -α	5’-AATATGTCCCCCAGACCAAAGA-3’	5’-GGCAGGCAAGTACCGGTTT-3’	FAM-CGCCTTGGCAAAAGACAGGACCA-TAMRA
Glutamate-cysteine ligase	5’CAGAGTATGGGAGTTACATGATTGAAG-3’	5’-TGTGTTGAACTCGGACATCGTT-3’	FAM-ACACCTGGCCAGCCGTACGGAG-TAMRA
Paraoxonase-1	5’-TGAGAGCTTCTATGCCACAAATG-3’	5’-CCATGACAGGCCCAAGTACA-3’	FAM-TTTTGCTGACCCATACTTACGGTCCTGG-TAMRA
Sulfiredoxin-1	5’-AATCCCCAACCCCTGACTTT-3’	5’-TGAACTGACCAGTGGAGACACAGT-3’	FAM-ACAAGGTTTCTTCAGCCCCGGTGC-TAMRA
GAPDH	5'- TGGCCTCCAAGGAGTAAGAAAC -'3	5'- GGCCTCTCTCTTGCTCTCAGTATC -'3	FAM- CTGGACCACCCAGCCCAGCAA-TAMRA

### Statistical analysis

Differences between obtained values (mean ± SEM, n = 6) were carried out by one way analysis of variance followed by the Tukey-Kramer multiple comparison. The differences were considered statistically significant at P<0.05.

## Results

Liver enzymes, ALT and AST levels in plasma were used as biochemical markers for the early acute hepatotoxicity. Rats fed with HCD for 6 weeks had significant increase in of AST and ALT levels (p<0.001) compared to control group. Rutin supplementation alone showed no significant changes in biochemical markers. However, administration of rutin in combination with HCD resulted in reversal of hepatic damage biomarker induced by HCD to normal values (Table 2). Lipid parameters of HCD fed rats including TG, TC and LDL levels were significantly increased in plasma by 48%, 89% and 67% respectively and significantly decreased the HDL levels by 17% compared to control group. Rutin supplementation in combination with HCD, significantly decreased TC and LDL levels compared to HCD group. On the other hand there is no effect on TG, TC, HDL and LHL was observed on the supplementation of RT alone (Table 2).

**Table 2:** Effect of High cholesterol diet, rutin and their combination on plasma levels of AST, ALT, TG, TC, HDL and LDL

**Group**	**AST (U/L)**	**ALT (U/L)**	**Triglycerides (mg/dl)**	**Total cholesterol (mg/dl)**	**HDL (mg/dl)**	**LDL (mg/dl)**
**Control (rat chow)**	35.64±1.52	22.76±0.84	63.03±5.99	66.67±4.25	29.91±1.32	48.32±2.32
**Rutin (0.2%)**	34.40±0.79	20.43±0.33	56.33±6.12	61.70±3.57	30.93±0.98	47.09±1.87
**High cholesterol diet**	53.30±3.07^*^	35.71±1.87^*^	93.30±4.94^*^	125.33±13.94^*^	24.87±1.54^*^	80.72±3.19^*^
**Rutin (0.2%) + HCD**	37.60±2.00^#^	23.10±1.25#	86.46±5.25^*^	98.06±7.06^*#^	26.86±1.35	71.50±2.93^*#^

Data were expressed as Mean±SEM. *&# indicate a significant change from control and HCD, respectively, at p < 0.05 using ANOVA followed by Tukey-Kramer as a post ANOVA test. Six animals were used in each group.

The effect of HCD, rutin and their combination on the oxidative stress biomarkers and indices of lipid peroxidation, MDA, H2O2 and GSH were shown in Table 3. The HCD feeding was resulted significant increase in liver MDA by 23 % and in plasma H_2_O_2_ by 354 %, and decrease in hepatic GSH level by 17% compared to the control group. Rutin administration in combination with HCD resulted in a significant decrease in the levels of MDA and H2O2 and increase the hepatic level of GSH compared to HCD group.

**Table 3:** Effect of High cholesterol diet, rutin and their combination on levels of MDA, GSH and H2O2

**Group**	**MDA (mmol/g)**	**GSH (nmol/100mg)**	**H2O2 (μM)**
**Control (rat chow)**	256±7.21	171.91±7.48	1.3±0.6
**Rutin (0.2%)**	260.50±7.49	170.53±4.06	1.1±0.43
**High cholesterol diet**	315.34±6.79^*^	143.07±2.16^*^	5.9±1.1*
**Rutin (0.2%) + HCD**	263.56±9.02^*#^	192.87±4.22^*#^	1.8±0.98^#^

Data were expressed as Mean±SEM. *&# indicate a significant change from control and HCD, respectively, at p < 0.05 using ANOVA followed by Tukey-Kramer as a post ANOVA test. Six animals were used in each group.

**Figure 1:**
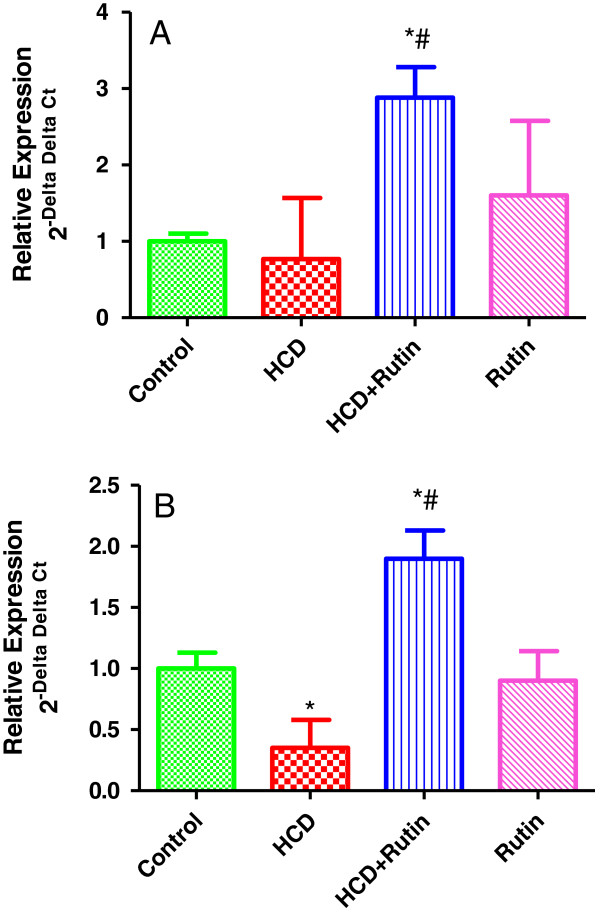
**Showing the Effect of HCD, rutin, and their combination on the expression levels of Glutathione peroxidase (A) and Glutathione reductase (B) in rat liver.** Data were presented as mean ± SEM (n = 6). * and # indicate significant change from control and HCD, respectively, at P < 0.05 using ANOVA followed by Tukey–Kramer as a post ANOVA test.

The present results showed an insignificant decrease by 23% in the expression of GPX gene and significant decrease by 65% in GR genes in rats fed with HCD compared to control group (Figure 1A & B). Interestingly, administration of rutin in combination with HCD resulted in a significant increase the expression of these genes by 245% and 441% compared to HCD group and by 166% and 90% compared to control group respectively.

**Figure 2:**
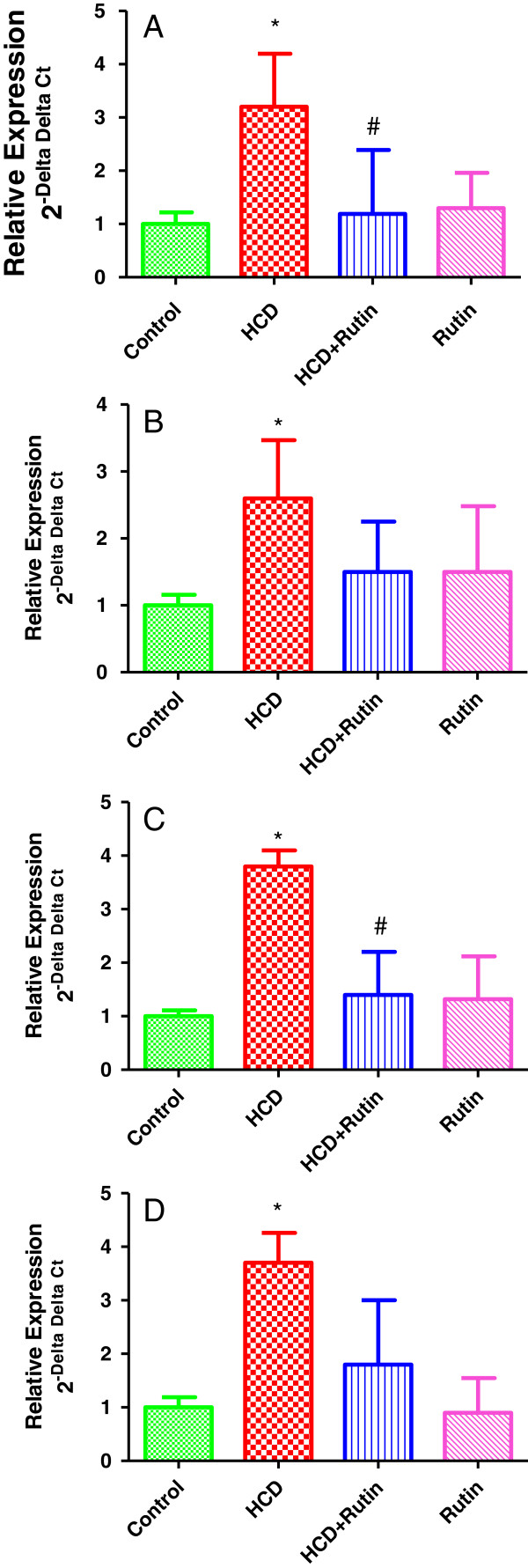
**Showing the Effect of HCD, rutin, and their combination on the expression levels of Glutathione S transferase α (A), paraoxonase-1 (B), sulfiredoxin (C) and glutamate-cystein ligase (D) in rat liver tissues.** Data were presented as mean ± SEM (n = 6). * and # indicate significant change from control and HCD, respectively, at P < 0.05 using ANOVA followed by Tukey–Kramer as a post ANOVA test.

The expression of Glutathione S transferase α, paraoxonase-1, sulfiredoxin and glutamate-cystein ligase were significantly increased by 220%, 160%, 250% and 230% respectively, in HCD fed rats compared to the control group (Figures 2A, B, C & D). The rutin supplementation with HCD resulted in significant decrease in the expression of Glutathione S transferase α, PON-1 and sulfiredoxin genes by 63% 130% and 54% respectively and an insignificant decrease in the glutamate-cystein ligase gene expression by 45% as compared with HCD group.

## Discussion

Obesity is a risk factor for many diseases such as cardiovascular [46] and liver diseases [3,4]. Rat models fed with HCD can be used as model of the human obesity syndrome [47,48]. The present study examined the hepatoprotective effect of rutin against hepatotoxicity induced by HCD in rat model and demonstrated that HCD caused hepatotoxicity through increasing plasma levels of liver enzymes ALT and AST. In agreement with earlier studies, the elevated ALT and AST levels are attributed to hepatic damage that may contribute to oxidative stress unbalance [49,50]. Rutin has reduced the oxidative stress in liver, kidney, and brain tissues of rats [51,52]. As a result of rutin supplementation, ALT and AST levels were lowered that led to decrease the hepatic damage caused by HCD feeding. The present results showed that rutin can protect hepatocyte against toxicity induced by HCD.

The persistent oxidative stress causes DNA mutation and increases fibroblastic activity, leading to liver cirrhosis and carcinoma. Previous study has demonstrated that rutin has a protective effect against HCD-induced liver cirrhosis [50]. Lipid alterations have been considered as contributory factors to oxidative stress in obesity resulted of increased in the production of ROS as well as reduced antioxidant enzymes [53,54]. Reactive oxygen species and lipid peroxidation products impaired the respiratory chain in hepatocytes through oxidative damage to the mitochondrial DNA. In the present study, HCD feeding resulted in increasing the levels of TG, TC and LDL and decreasing in HDL as compared with control group; our finding was in agreement with other studies [55,56].

High cholesterol diet leads to dyslipidemic syndrome and hyperlipidemia that characterized by increasing in TG and decreased in HDL-Cholesterol. [57]. Dyslipidemic syndrome produced anti-inflammatory effects by inhibiting the expressions of proinflammatory cytokines [58,59]. In the present study, rutin-supplement attenuated HCD-induced hepatotoxicity by lowering the concentrations of TC, TG and LDL. Similarly, rutin lowers the lipid components in the serum of hyper-cholesterolemic rats, probably by reducing the activity of 3-hydroxy-3-methyl-glutaryl-CoA reductase [60]. This may be explained on the basis that rutin has a strong ability to chelate multivalent metal ions, especially zinc, calcium and iron [39].

Lipid peroxidation is characterized by imbalance between oxidant-antioxidant and ROS are thought to be a component of obesity-induced pathology [61,62]. The data of this study showed that HCD increased lipid peroxidation in hepatic tissue as expressed by increased tissue levels of MDA, this will cause an increased accumulation of H_2_O_2_ which could further stimulate lipid peroxidation. The present results were convenient with earlier studies [62,63] showed that obesity is an independent risk factor for increasing lipid peroxidation and decreased activity of cytoprotective enzymes. Damage, at the cellular level by oxidative stress, is attenuated by antioxidant enzyme such as PON-1, GSHPx, GPx, GR and Glutathione S transferase α, sulfiredoxin and glutamate-cystein ligase. When the balance between ROS production and antioxidant defense is lost oxidative stress occurred through a serious of events deregulates the cellular functions leading various pathological conditions [26].

The GSH antioxidant system plays critical role in the detoxification process of liver and is involved in overcoming various hepatotoxins-induced liver injuries [64]. The increasing GSH levels can protect cells against oxidative damage, while depleting cellular GSH can promote such injury [65,66]. Our results showed that GSH level was decreased in HCD fed rats compared to control which was in agreement with other [67]. The cellular roles of GR have been broadened in various physiological phenomena, especially cellular response against many kinds of stresses by reducing glutathione disulfide (GSSG) to the sulfhydryl form GSH which is an important cellular antioxidant [68]. Glutathione peroxidase is a selenoenzyme, which catalyzes the reduction in hydrogen peroxide to H_2_O and oxidizing GSH into GSSG [69]. Down-regulation of GR results in cellular GSSG content increase, and reduction of GSH/GSSG ratio is involved in many responses against oxidative stress. Our results showed decrease in GR and GPx genes expression in liver tissues of HCD fed rats and were in agreement with others [70,71]. Rutin supplementation led to increase the expression of these genes in liver tissues. These data showed that HCD not only increase the free radical formation but also decrease its ability to detoxify ROS, which lead to hepatocellular damage [72].

Paraoxonase-1 (PON-1), an enzyme with lactonase and esterase activities, is synthesized mainly by the liver [73,74] and it plays a role in the regulation of oxidative stress, fibrosis and hepatic cell apoptosis in chronic liver diseases. [75]. The present results showed that the HCD feeding significantly overexpressed the expression of PON-1 in liver tissues. This increase in the expression enhanced the sensitivity to liver damage development. The increasing in PON-1 hepatic expression in chronic hepatitis and liver cirrhosis, probably as a response to the enhanced oxidative stress observed in the earliest stages of these diseases [75]. Our results were disagreement with Zhang et al., who proposed that PON-1 over expression provides strong protection against the development of experimental liver disease [76]. Rutin supplementation led to decrease the expression of PON-1 gene in liver tissues, and this attributed to its effect as an antioxidant and reduced oxidative stress in plasma, liver and other organs [51,77]. The present finding was in agreement with recent study who found that rutin administered to High fat diet fed rats attenuated the diet-induced metabolic syndrome, NASH, and cardiovascular abnormalities [78].

Glutamate-cysteine ligase catalyzes the biosynthesis of cellular GSH and is considered one of antioxidant system for counteracting ROS produced during oxidative stress injury. Sulfiredoxin-1 (Srx1), an antioxidant, contains a C-terminal cysteine residue that is highly conserved and crucial for its antioxidant function [79,80]. It plays a key role in cellular responses to oxidative stress by restoring the activity of over-oxidized peroxiredoxins [80,81]. The results of the present study show that Srx1, GCL and GSTα expressions are selectively up-regulated in liver tissues of rat fed with HCD. Reactive oxygen species elicit many different responses depending upon the severity of the damage and the duration of the exposure [82].

Low doses of ROS activate c-jun N-terminal Kinase (JNK) transiently thereby promoting cell proliferation. However, persistent JNK activation due to severe oxidative stress ultimately causes cell death via activation of pro-apoptotic signaling pathway [83,84]. In the current study the activation of GSTα was due to the modulations of GST levels on JNK activation by formation of GSTα-JNK complex integrity in sequence to inhibit its activation. Our results was in agreement with other studies [85,86], who shows that, besides detoxification of GST-α, it plays an important role in signaling events by modulating stress cell signaling kinases in particular through inhibition of JNK activation. The upregulation of GCL in our data was in agreement with Nishiya et al., who found that, marked upregulation of GCL gene in rats treated with tienilic acid induced hepatotoxicity [87]. Similarly, Wu and his colleague found that Cd-induced oxidative stress increased the mRNA expression of GCL with graded Nrf2 activation. [88]. However, the present data is disagreement with Wang and his colleagues; they found that the downregulation of GCL is confirmed the damage of liver cells in mice treated with *Dioscorea bulbifera* rhizome [89]. The up-regulation of Sxr1 to maintains the balance between H2O2 production and elimination and then protects liver cells from apoptosis; our finding was in agreement with previous study [90]. In the present study rutin administration reversed the changes induced by HCD feeding in rats to normal levels probably by reducing oxidative stress and inflammation in liver tissue.

## Conclusion

The high-cholesterol diet induces symptoms of metabolic syndrome in rat including hepatotoxicity with alteration in the oxidative stress markers. Rutin reversed the changes induced by HCD probably by reducing the oxidative stress and inflammation in the liver. Therefore the administration of rutin may be used as an antioxidant in decreasing the hepatic stress in humans as a treatment.

## Competing interests

The authors declare that they have no competing interests.

## Authors’ contributions

SA, HA and MA, equally participated in the dietary protocol design of the experimental groups and performed the biochemical assays and their statistical analysis and shared in drafted the manuscript. MH performed all the molecular studies and their statistical analysis and shared in draft the manuscript. AA, MS, OA and KA shared in the draft the manuscript. All authors read and approved the final manuscript.

## Pre-publication history

The pre-publication history for this paper can be accessed here:

http://www.biomedcentral.com/1472-6882/13/136/prepub
